# Conformational Changes of the Receptor Binding Domain of SARS-CoV-2 Spike Protein and Prediction of a B-Cell Antigenic Epitope Using Structural Data

**DOI:** 10.3389/frai.2021.630955

**Published:** 2021-03-25

**Authors:** Sangeeta Khare, Marli Azevedo, Pravin Parajuli, Kuppan Gokulan

**Affiliations:** Division of Microbiology, National Center for Toxicological Research, United States-Food and Drug Administration, Jefferson, AR, United States

**Keywords:** spike protein, SARS–CoV−2, receptor binding domian, conformational epitope, viral infection

## Abstract

COVID-19, the illness caused by the SARS-CoV-2 virus, is now a worldwide pandemic with mortality in hundreds of thousands as infections continue to increase. Containing the spread of this viral infection and decreasing the mortality rate is a major challenge. Identifying appropriate antigenic epitopes from the viral proteins is a very important task for vaccine production and the development of diagnostic kits and antibody therapy. A novel antigenic epitope would be specific to the SARS-CoV-2 virus and can distinguish infections caused by common cold viruses. In this study two approaches are employed to identify both continuous and conformational B-cell antigenic epitopes. To achieve this goal, we modeled a complete structure of the receptor binding domain (RBD) of the spike protein using recently deposited coordinates (6vxx, 6vsb, and 6w41) in the protein data bank. In addition, we also modeled the RBD-ACE2 receptor complex for SARS-CoV-2 using the SARS-CoV RBD-ACE2 complex (3D0J) as a reference model. Finally, structure based predicted antigenic epitopes were compared to the ACE2 binding region of RBD of SARS-CoV-2. The identified conformational epitopes show overlaps with the ACE2-receptor binding region of the RBD of SARS-CoV-2. Strategies defined in the current study identified novel antigenic epitope that is specific to the SARS-CoV-2 virus. Integrating such approach in the diagnosis can distinguish infections caused by common cold viruses from SARS-CoV-2 virus.

## Introduction

The first incidence of COVID-19 was reported in China in late December 2019. The COVID-19 infection caused symptoms of severe acute respiratory syndrome, severe respiratory illness, fever, and pneumonia (Huang et al., 2020; WHO, 2020; Zhou et al., 2020; Zhu et al., 2020). The causative agent for this disease is designated as SARS coronavirus-2 (SARS-CoV-2). The ongoing SARS-CoV-2 illness was declared a global pandemic with mortality in the hundreds of thousands and infections continuously increasing every day. The rate of mortality is much higher in individuals who have preexisting health issues that include cancer, hypertension, diabetes, coronary heart disease, liver disease, and pre-existing digestive diseases. The genetic material of SARS-CoV-2 is a single-strand positive sense RNA, and the so-called spike protein protrudes from the envelope layer. The genome analysis reveals that SARS-CoV-2 belongs to the same subgenus of SARS-CoV (Sarbecovirus), thus it has been classified as a new member of betacoronavirus genus (Xu et al., 2020). The hosts for coronaviruses are birds and mammals, but they have crossed the species barrier and evolved as a zoonotic pathogen (Anthony et al., 2017; Zhou et al., 2020). Both SARS-CoV and SARS-CoV-2 spread through human to human contact. However, the rate of viral transmission and infectivity of SARS-CoV-2 is far greater compared to SARS-related coronavirus (Chen et al., 2020).

Like SARS-CoV, the spike protein of SARS-CoV-2 also initiates binding with ACE2 receptors on the host cells through its spike protein and it replicates in primary airway epithelial cells, lungs, and the gastrointestinal system (Phan, 2020). Both SARS-CoV and Middle East Respiratory Syndrome-CoV (MERS-CoV) are etiological agents for an acute respiratory syndrome that has caused mortality in the last two decades (Gralinski et al., 2018). In 2003, SARS-CoV caused a mortality rate of 9.6% (774/8,076) (WHO, 2015), whereas in 2013 MERS-CoV infection caused a mortality rate of around 34.4% (858/2,494) (WHO, 2015, 2019). As of April 27, 2020, SARS-CoV-2 caused infection in 3,029,736 individuals and the mortality of 209,244 individuals (7% CMR). It is evident that the SARS-CoV-2 pathogenesis might be different from that of SARS-CoV and MERS-CoV coronaviruses. Recent pathological studies reveal that SARS-CoV-2 causes alveolar damage, edema, type-II hyperplasia pneumonia, inflammation, and acute respiratory distress syndrome (ARDS) (Xu et al., 2020). However, these viruses use the spike protein to enter host cells. The spike protein is composed of S1 and S2 domains. The S1 domain plays an essential role in the initial binding with host cell receptors, whereas the S2 domain is responsible for the fusion and entry of the virus into host cells (Gui et al., 2017; Tortorici and Veesler, 2019). The structure of the spike protein of SARS-CoV-2 was published by cryo-electron microscopy (cryo-EM) (Walls et al., 2020; Wrapp et al., 2020). The solved structures provide evidence that the spike proteins exist in two conformations [closed (pre-fusion) and open (post-fusion)]. It is evident that the spike protein undergoes conformational changes to interact with the ACE2 receptor. This manuscript focuses mostly on the conformational changes between the open and closed structure of the spike protein of SARS-CoV-2, and in particular to the receptor binding domain (RBD).

The structural information reveals that the S1 domain is primarily composed of β-strands in the center (Wrapp et al., 2020). Both sides of β-strands are surrounded by several α-helices, and it folds into a V shaped architecture. Particularly, the structural fold shows that the receptor binding domain (RBD) packs against the N-terminal region. These two regions are connected by a hinge loop that allows them to fold against each other. In addition, the RBD is covered with a flexible loop conformation on the top of its globular head. The S2 domain is mostly composed of both α-helices and β-strands and is highly glycosylated. Similar to S1, the β-strands in S2 also occupy the center of the core of trimer and are surrounded by α-helices on the outside. These two domains are connected by a long flexible loop that provides these domains movement and flexibility. Three monomeric subunits of spike protein assemble and form a functional unit, which is attached to the envelope protein through the transmembrane region (C-terminal region). Upon assembly, the RBD occupies the top of the homotrimer and forms a globular head and protrudes from the viral cell surface. The N-terminal region (1–300 amino acids) of S1 occupies just below the RBD 42 Å away from the core of the homotrimer. It is important to note that among all coronaviruses, amino acids of the S1 domain are highly variable and few residues are conserved. The N-terminal portion of the S1 domain (residues 1–350) is highly glycosylated, whereas the S2 domain is intermittently glycosylated within the entire region that initiates viral fusion inside the host cells. Unlike the S1 domain, residues at the S2 domain are highly conserved in various coronaviruses.

## Methods

### Homology Modeling of SARS-CoV-2 Structure

Atomic coordinates for SARS-CoV-2 spike protein are available in the Protein Data Bank (PDB), and 6vsb and 6vxx coordinates were downloaded from the PDB http://www.rcsb.org/pdb. The crystal structure of the RBD of SARS-CoV-2 complexed with monoclonal antibody has been recently deposited (PDB 6w41) (Yuan et al., 2020) in PDB. This antigen-antibody complex structure was solved at a higher resolution with a complete structure of the RBD of SARS-CoV-2. The RBD was extracted from the complex structure (PDB 6w41) for structural superposition on top of the RBD from the 6svb and 6vxx coordinates to get a complete structural fold of RBD of SARS-CoV-2. Earlier, the crystal structure of a chimeric molecule of the ACE2 receptor complexed with RBD of SARS-CoV (3D0J) was reported (Li, 2008). The 3D0J coordinate was downloaded from the PBD as a reference model. The chimeric molecule of the ACE2 receptor complexed with the RBD of SARS-CoV was used as a reference model to build RBD-ACE2 complex for SARS-CoV-2. The modeled binary complex structure was used for analyzing the interaction between ACE2 with RBD complete structure. Before structural comparison of this complex, these modeled structures were energy minimized in the Insight II suite using Discovery (Accelrys). Win coot, Pymol, chimera, and Insight II suite were used for conducting structural analysis and comparison and generating figures.

### Conformational and Linear Antigenic Epitope Prediction

Genome sequence retrieval: We downloaded SARS genome sequence and protein sequence for BAT, SARS-61-TW. SARS-Wahun-2, SARS-USA, SARS-ITA, and SARS-Ind from NCBI GenBank. Multiple sequence alignment was performed in CLUSTAL-OMEGA online software to identify the sequence homology, % of conserved and non-conserved residues (http://www.ebi.ac.uk/support/sequence-alignment). Preceding section describes the complete homology modeling and building of SARS-CoV2 spike protein structure using various PDB coordinates. The modeled spike protein was energy minimized and inspected by structural superposition with Cryo-EM structure of SARS-CoV2 spike protein. To identify structure based conformational antigenic epitope prediction, we used two different bioinformatic tools based on the surface accessibility and propensity (Haste Andersen et al., 2006; Ponomarenko et al., 2008). To identify linear B-cell antigenic or continuous B-cell epitopes online bioinformatic tools (http://tools.iedb.org/bcell) was used. The antigenic epitopes were predicted using several methods that utilize various parameter. The protein sequence was submitted to predict the antigenic epitope based on the criteria that include beta-turn predictions, surface accessibility, flexibility, and hydrophilic scale (Chou and Fasman, 1978; Hopp and Woods, 1983; Emini et al., 1985; Karplus and Schulz, 1985; Parker et al., 1986; Kolaskar and Tongaonkar, 1990; Lins et al., 2003; Jespersen et al., 2017). For example, Emini predicts the protein sequence based on surface accessibility, whereas BepiPred-2.0 server predicts B-cell epitope from the protein sequence using Random Forest algorithm and trained antigenic epitope from the crystal structure. Both conformational and linear antigenic epitopes were further analyzed for allergenicity using Allercatpro (https://allercatpro.bii.a-star.edu.sg/).

## Results and Discussion

### Structural Analysis of Open and Closed Conformation

**Figure 1:**
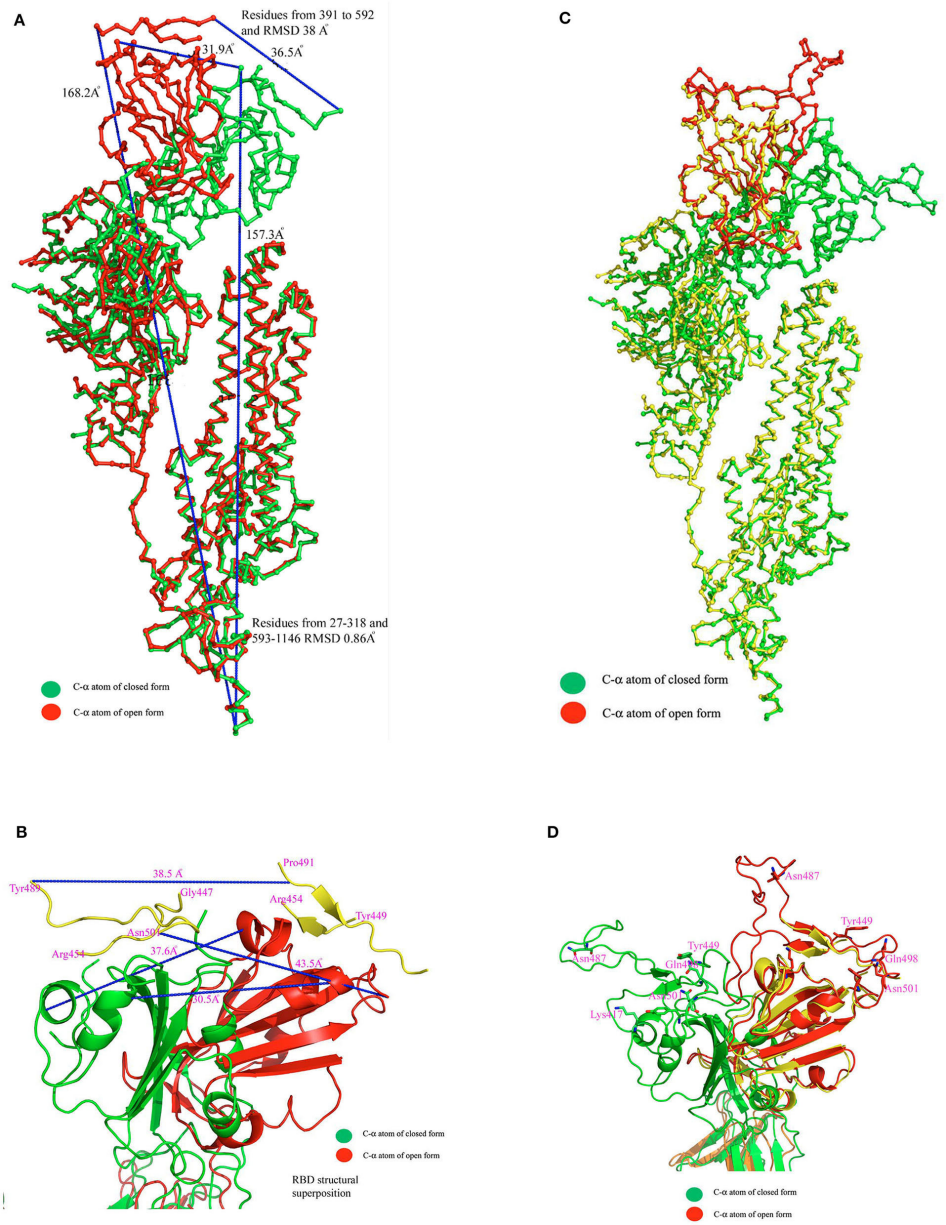
**(A)** C-α atom superposition of 6vsb with 6vxx coordinates. This alignment shows C-α atoms from residues 27–318 and 563–1146 aligns on top of each other with root mean square deviation (rmsd) 0.86 Å. **(B)** Magnified cartoon diagram that shows only RBD of both structures with superposition and RMSD between these two structures is ~38 Å. The globular head shows the missing loops region in both structures and part of the region is shown in yellow color. Color code: Green color-closed form and red color-open form. **(C)** Structural superposition of the RBD of 6w41 coordinates on top of RBD of 6vxx and 6vsb. **(D)** Cartoon diagram shows the superposition of the RBD of 6w41 coordinates on top of both structural forms. Color code: Green color-closed form, red- (complete structure of RBD from pdb 6w41), and yellow (RBD lack loops) the open form. The distance between these two structures are also shown in dotted lines.

Recently the structure of SARS-CoV2 spike protein has been solved by two groups using cryo-EM (Walls et al., 2020; Wrapp et al., 2020). The McClellan laboratory has shown that all subunits of trimeric complex are either in closed conformation (pre-fusion stage) or open conformation (post fusion) of the functional unit (Wrapp et al., 2020). Walls et al. (2020) have shown that one subunit is in open form and the other two subunits are in closed form. Both structures reveal that a homotrimer is the functional unit for binding with ACE2 receptors. While comparing the structural superposition of these two structures, C-α atoms from residues 27–318 and 593–1,273 align very well between the open and closed conformation with root mean square deviation (rmsd) 0.86 Å. In contrast, C-α atoms from residues 319–592 did not align well and show major structural differences between these two conformations with a rmsd of ~38 Å (Figure 1A). Both cryo-EM structures provided a wealth of information but lack several connecting loop regions in the S1 domain. Particularly, the missing loop regions are located near the globular head, which is essential for ACE2 receptor binding (Figure 1B). In order to get complete structural information of the RBD of SARS-CoV-2, the crystal structure of the SARS-CoV-2 RBD domain of 6w41 coordinate was superimposed on top of both the closed and open form of SARS-CoV-2 spike protein structures individually to get the complete structural information of RBD (Figure 1C). Obtaining complete structural information of the RBD will provide a better understanding of the structural rearrangement between these two forms and it can also provide a clear picture of the interaction with the ACE2 receptor molecule (Figure 1D).

**Figure 2:**
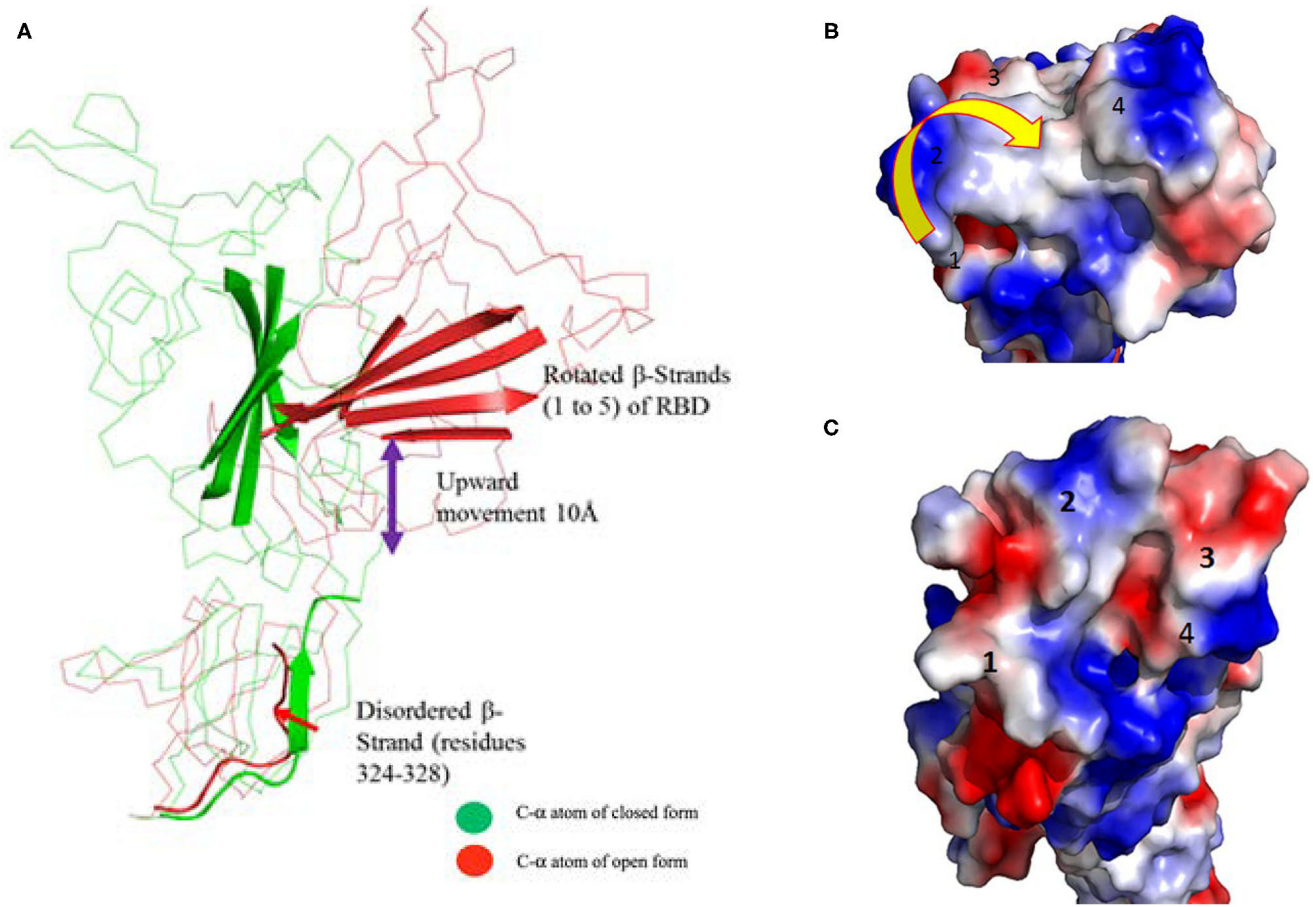
**(A)** Cartoon diagram shows that residues 324–328 form a loop instead of β-strand in open conformation. This cartoon representation shows the β-strands of RBD domain movements during the conformational change. The figure also shows that during open conformation the entire domain moves 10 Å upwards (shown by blue arrow) and β-strands rotational angles changes from 75 to 110° (see Table 1 for detail information). Color code: Green-closed form and Red-open form. **(B,C)** Electropotential surface of the RBD for closed and open conformations, respectively. It also shows structural rearrangement leads to change in the electropotential surface, which are highlighted by numbers (1 to 4). The rearrangements occur at clockwise direction during structural change. Right side top figure is for closed form and bottom is for open form.

The superimposed model had a complete structure of the RBD of SARS-CoV-2, so it was used for further structural analysis. The RBD is connected by a long loop (319–337) containing a short β-strand (residues from 324–338) in the middle of the closed form (Figure 2A). In contrast, no ordered β-strand is found in the open form and part of the region is disordered. The alignment also reveals a 2.6 Å rmsd between them at amino acid position 319. The structural movement is gradually increased until the globular head as shown in Figures 1A,B. The rmsd at the globular head is ~38 Å between the open and closed conformation. In addition, in open conformation the RBD moved up and outward from the core structure in comparison with closed conformation. The RBD is composed of five anti-parallel β-strands (named as β1 to β5) (Table 1) in the middle and surrounded by several α-helices at the outside. The structural movement of each individual β-strand of RBD is further compared and analyzed for upward movement and rotational angle as well (Figure 2A). The table shows the structural movement of individual β-strands of the RBD between these two forms. We also analyzed the electropotential surface between the closed and open conformation of the RBD of SARS-CoV-2. The structural rearrangement leads to change in the electropotential surface on the globular head. Particularly, the globular head region rotates in a clockwise direction as shown by numbers (labeled as 1 to 4) in Figures 2B,C.

**Table 1:** Conformational changes and rotational angles of β-stands of RBD between open and closed conformation.

**β-strand**	**Residues closed form**	**Residues open form**	**Rotational angle^**°**^**	**Upward movement Å**	**Distance from starting point to end Å**
β1	354–358	354–358	75	3.8	6.9 & 15.6
β2	375–379	375–380	109	9.7	11.1
β3	393–403	391–403	90	3.8	3.6 & 31.3
β4	431–437	431–437	102	9.9	10.2
β5	507–516	507–516	107	12.9	12

### Interaction of RBD With ACE2 Receptor

**Figure 3:**
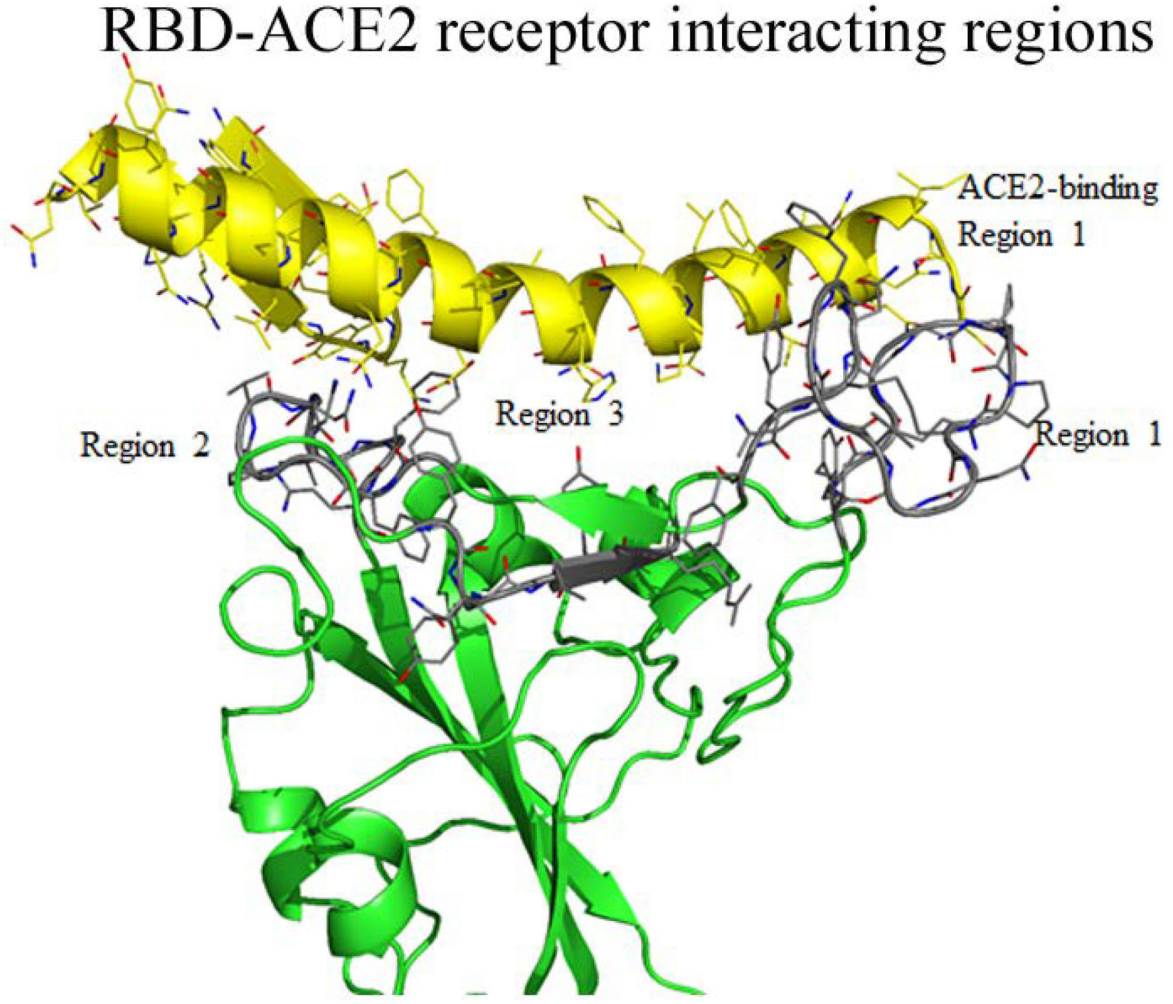
Interaction between regions of RBD of SARS-CoV-2 and the ACE2 receptor molecule. The RBD globular head forms a bowl-shaped structure and both ends are covered with elevated loops. Residues from these loops are positioned to interact with the ACE2 receptors and labeled as ACE2 receptor binding region 1 to 3 and highlighted with gray color. The interacting residues of ACE2 receptor also labeled as region 1 to region 3.

Earlier, the crystal structure of the RBD of SARS-CoV complexed structure had been deposited in PDB (PBD 3D0J). To understand the possible interaction between the RBD of SARS-CoV-2 with the ACE2 receptor we used 3D0J coordinates as a reference model and the RBD-AEC2 complex structure was modeled for SARS-CoV-2. This complex structure was further energy minimized and validated for bond interaction to inspect the clash in the binary complex. The minimized structure reveals the interaction regions between the RBD and the ACE2 receptor. In the RBD, interacting residues are clustered into three regions (Figure 3). Residues (474–488) from the first region are close enough to interact with the N-terminal region (residues 21–35) of the ACE2 receptor. Additionally, in this region two cysteine residues (480 and 488) form a disulfide bond that makes a stable loop. Residues from the second region (496–506) are in close proximity to the ACE2 receptor. This arrangement enables the ACE2 receptor to interact with two different parts of the spike protein that includes residues from 41 to 43 from the N-terminal region and residues from the 351–355 of the connecting loop of β-strands. The third region of RBD (residues 449–453) is in suitable position to interact with residues from 34 to 38 of the N-terminal region of the ACE2 receptor. Overall residues from 449–453 to 489–499 of the RBD form a base of the concave surface, which is surrounded by an elevated loop on both sides (Figure 3). These elevated loops consist of 8 to 9 amino acids and they are close enough to interact with the ACE2 receptor. The modeled structure reveals that region-1 and region-2 are located on the outer surface and exposed to solvents. Region-3 is placed somewhat in the interior and occupied next to region-1 and its exposure to solvents is limited. The residues of these three interacting regions were compared with the SARS-CoV RBD complexed with the ACE2 receptor (3D0J coordinates). The amino acid sequence comparison shows that there are conserved amino acids among them (Table 2).

**Table 2:** Sequence comparison between modeled SARS-CoV-2 RBD-ACE2 complex and chimeric ACE2-RBD of SARS-CoV.

Region-1	SARS-CoV (463–477)	SPDGKPCTP- PALNCY
	SARS-CoV-2 (474–489)	QAGSTPCNGVEGFNCY
		..... **.. _... ***
Region-2	SARS-CoV (478–495)	WPLNDYGFYTTTGIGYQPYR
	SARS-CoV-2 (490–509)	FPLQSYGFQPTNGVGYQPYR
		***.. ***.. *. *. ******
Region-3	SARS-CoV (433–441)	TGNYNYKYR
	SARS-CoV-2 (446–454)	GGNYNYLYR
		. *****. **

*Indicates the conserved of residues between SARS-CoV and SARS-CoV-2, _ indicates amino acid insertion and … indicates non-conserved residues.

*Red indicates hydrophobic residues, and are either conserved or similarly charged*.

*Green and blue indicates hydrophilic residues, and are either conserved or similarly charged*.

### Structure Based Antigenic Epitope Prediction

Recent studies have shown that the N-terminal region of the S1 domain of the SARS-CoV-2 is highly variable in comparison with SARS-related coronavirus. However, the SARS-CoV-2 genome sequence reveals 79.5% sequence identity with SARS-CoV at the nucleotide level (Zhou et al., 2020), which indicates the close resemblance between them. The spike protein of the SARS-CoV-2 has 76.47% of sequence identity with the spike protein of SARS-CoV (Zhou et al., 2020). The genome from different geographical origins of SARS-CoV-2 isolates have 99.9% of sequence conservation among them (Simmonds et al., 2020). Earlier, the chimeric structure of the ACE2 receptor complexed with the RBD of the SARS-CoV identified critical residues for viral infection (Li, 2008). Hua et al. (2004) identified antigenic epitopes from the spike protein of SARS-CoV and only a few peptides reacted with monoclonal antibodies raised against a fusion peptide (Hua et al., 2004). One of the antigenic epitopes was identified at residues from 446 to 461 in SARS-CoV and the equivalent region (residues 459–474) is partially conserved in SARS-CoV-2 (Table 3). Furthermore, antigenicity properties for this partially conserved region in (residues 459–474) SARS-CoV-2 is unknown. Secondly, this epitope region is found to be away from the ACE-2 receptor binding region. The other predicted antigenic epitope is from the S2 domain of SARS-CoV (residues 779–809), which is highly conserved in SARS-CoV-2 as well (residues 796–826) (Table 3). In another study monoclonal antibodies were generated for the whole SARS-CoV virus and for linear peptide antigenic epitopes; however, the monoclonal antibody generated to the whole virus cross reacted with both linear epitopes as well as with a conformational epitope (Berry et al., 2010). This study indicates that the RBD antigenic epitope is most likely exposed to the viral surface (Berry et al., 2010). In this study we used the spike protein structure containing a complete structure of the RBD to identify conformational epitopes.

Table 3Antigenic epitopes from spike protein of SARS-CoV in comparison with SARS-CoV-2 of spike protein.

**Table:** 

**Start**	**End**	**Sequence**	**No. of residues**
329	363	FPNITNLCPFGEVFNATRFASVYAWNRKRISNCVA	35
369	393	YNSASFSTFKCYGVSPTKLNDLCFT	25
404	426	**GDEVRQIAPGQTGKIADYNYKLP**	23
440	501	**NLDSKVGGNYNYLYRLFRKSNLKPFERDISTEIYQAGSTPCNGVE GFNCYFPLQSYGFQPTN**	62
516	536	ELLHAPATVCGPKKSTNLVKN	21

**Table:** 

Antigenic epitope from S1 domain	SARS-CoV 446–461	GKLRPFERDISNVPFS
	SARS-CoV2 446–461	SNLKPFERDISTEIYQ
Antigenic epitope from S2 domain	SARS-CoV 779–809	GGFNFSQILPDPLKPTKRSFIEDLLFNKVT
	SARS-CoV2 796–826	GGFNFSQILPDPSKPSKRSFIEDLLFNKVT

*The red, blue, and purple color indicates the conserved residues of hydrophobic, acidic and basic residues, respectively. The green color indicates similar charged residues*.

Studies have shown that SARS-CoV-2 infected individuals have higher levels of antibodies. The secreted antibody levels are much higher in ICU patients compared to non-ICU patients (Huang et al., 2020). It is evident that cytokines probably play a major role in disease progression and severity. The increased viral replication and viral load during early stages of the infection have been correlated to an increase in apoptosis of epithelial and endothelial cells and vascular leakage, which further causes the secretion of proinflammatory cytokines through the activation of immune cells (Yang, 2020). Humoral immune response is essential for viral clearance, in particular antiviral neutralizing antibodies. Animal studies have shown that the SARS-CoV spike protein antibodies caused pulmonary injury by altering inflammatory immune responses (Liu et al., 2019). Vaccine induced pulmonary injury was also reported in Chinese rhesus monkeys, mice, and African green monkeys upon immunization of the S-protein of SARS-CoV (Bolles et al., 2011; Tseng et al., 2012; Liu et al., 2019). SARS-CoV infected individuals who developed a humoral response against the S-protein at an earlier stage of infection had higher mortality compared to patients who developed a humoral response at a later stage (Zhang et al., 2006). Currently several approaches have been employed to develop antivirus immune response against the SARS-CoV-2 by various researchers and companies. These approaches include inactivated virus, viral vectors, nucleic acids, recombinant proteins, and peptide-based antigenic epitopes. Antibody dependent enhancement (ADE) of viral pathogenesis has also been reported to other viral diseases (Takada and Kawaoka, 2003; Haslwanter et al., 2017). In the case of SARS-CoV-2 infection, identifying neutralizing antigenic epitopes which lack ADE is very important. Despite the promising results of the vaccine clinical trials, experts are very cautious for the complete eradication of the infection and making plea to be cautious about the cyclical illness that might stays with us, much like the flu (Corbett et al., 2020a,b; Forbes, 2020; Ho, 2020; Jackson et al., 2020).

**Figure 4:**
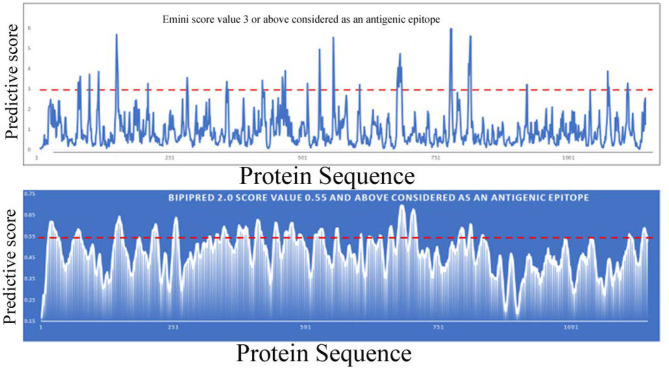
Linear B-cell epitope prediction by various bioinformatics tools [Emini (upper panel), BepiPred-2.0 (lower panel)].

Bioinformatic tools and a homology modeling approach have been employed here to identify an antigenic epitope from the spike protein of SARS-CoV-2. Initially, the spike protein sequence is used to predict continuous B-cell epitopes based on several criteria that include beta-turn predictions, surface accessibility, flexibility, and hydrophilic scale (Chou and Fasman, 1978; Hopp and Woods, 1983; Emini et al., 1985; Karplus and Schulz, 1985; Parker et al., 1986; Kolaskar and Tongaonkar, 1990; Lins et al., 2003; Jespersen et al., 2017) (http://tools.iedb.org/bcell). Most of the programs predictive scores are much higher in the S1 domain compared to the S2 domain. In addition, the number of predictive epitopes is also higher in the S1 domain, suggesting that more antigenic epitopes are present in the S1 domain. Furthermore, the S1 domain is more accessible to solvents or composed of a higher number of hydrophilic residues than the S2 domain. Overall these analyses indicate that the S1 domain is more accessible to be recognized by an antibody molecule or immune cells (B-cells and T-cells or other immune cells) (Figure 4). Earlier, it was shown that only a small percentage of B-cell epitopes are continuous epitopes, whereas the majority of B-cell epitopes are conformational epitopes (Van Regenmortel, 2009). To predict conformational epitopes, it is required to have prior knowledge about the structural information of that particular molecule.

To predict conformational epitopes from the spike protein of SARS-CoV-2 we used a current modeled structure (6vxx modeled with a complete coordinate containing RBD) containing complete structural information of the RBD. In this study the model is used in two different bioinformatic tools to predict conformational antigenic epitopes based on the surface accessibility and propensity (Haste Andersen et al., 2006; Ponomarenko et al., 2008). Park et al. (2020) recently reported that SARS-CoV neutralizing antibodies are generated to conformational epitopes, whereas in MERS-CoV the neutralizing antibodies are generated to linear epitopes. The surface accessibility is an important criterion for interaction with a partner protein such as a receptor or antibody molecule. Both programs predicated three conformational antigenic epitopes in the S1 domain and 1 antigenic epitope from the S2 domain (Table 4). The predicted conformational antigenic epitopes of the RBD are compared with binding regions of the RBD-ACE2 complex of SARS-CoV (Li, 2008), as well as the predicted binding region of the RBD-ACE2 binary complex (SARS-CoV2 in this study). The predicted antigenic epitope region is consistent with the earlier reported RBD-ACE2 receptor complex of SARS-CoV (Li, 2008). We also analyzed allergenicity for both conformational and linear antigenic epitope using Allercatpro. The output results revealed that all predicted antigenic peptides lacked allergenicity property. Additionally, we have also evaluated furin cleavage site for antigenic peptides; none of the antigenic epitopes overlaps with furin cleavage site (Gioia et al., 2020).

**Table 4:** Representative predictive antigenic epitopes.

**Start**	**End**	**Sequence**	**No. of residues**
333	339	TNLCPFG	7
359	371	SNCVADYSVLYNS	13
376	385	TFKCYGVSPT	10
430	435	**TGCVIA**	6
488	495	**CYFPLQSY**	8
505	527	YQPYRVVVLSFELLHAPATVCGP	23
**Start**	**End**	**Sequence**	**Score**
879	894	**AGTITSGWTFGAGAAL**	0.97
594	609	GVSVITPGTNTSNQVA	0.95
257	272	GWTAGAAAYYVGYLQP	0.95
1,112	1,127	PQIITTDNTFVSGNCD	0.95
245	260	HRSYLTPGDSSSGWTA	0.92
1,180	1,195	QKEIDRLNEVAKNLNE	0.92
476	491	**GSTPCNGVEGFNCYFP**	0.91

*Orange color highlighted, and green color bolded sequences are predicted by two or three methods*.

**Figure 5:**
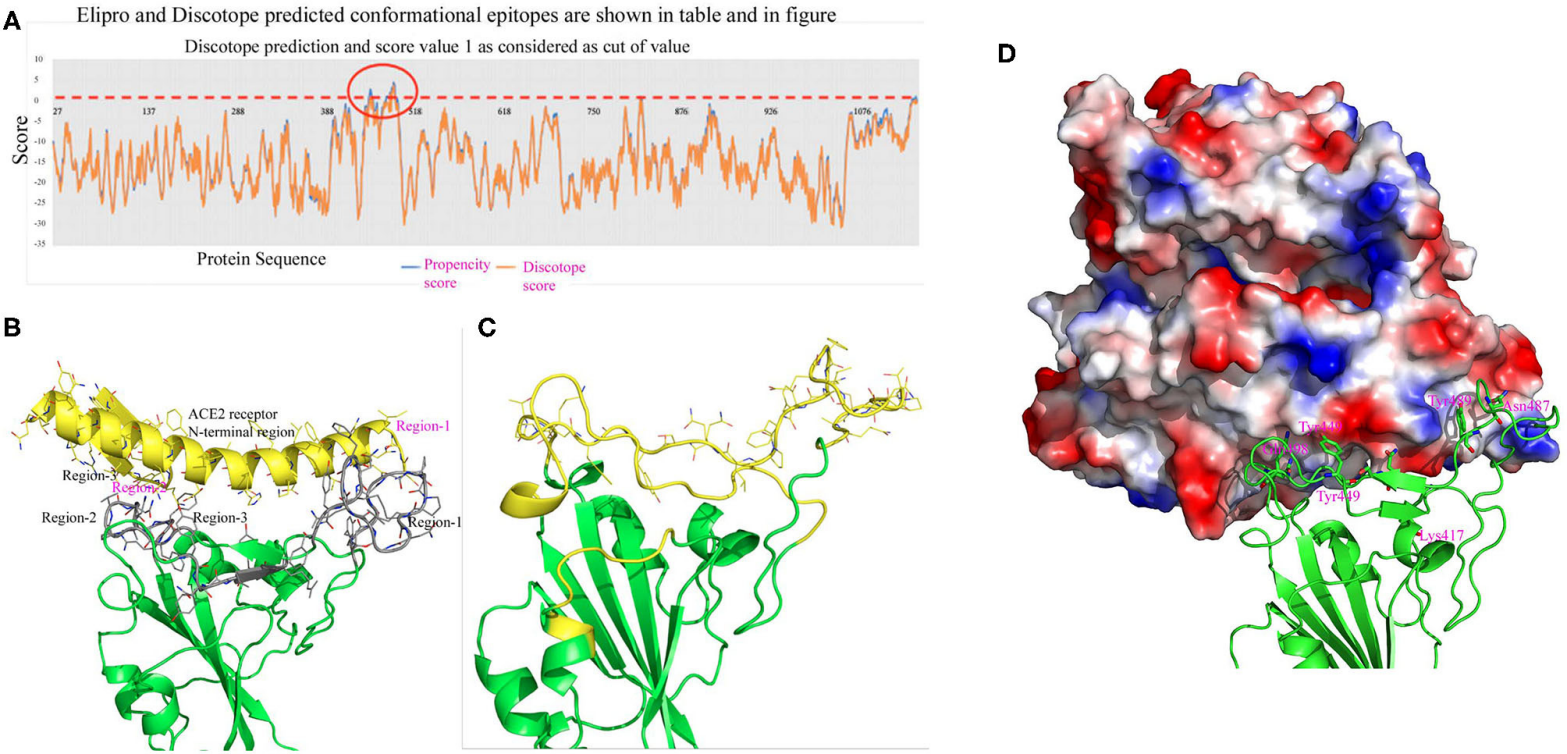
**(A)** This figure shows conformational epitopes as predicted by Elipro, Discotope and SEPPIA-3. **(B)** Predicted ACE2 interacting regions are shown in gray color. The ACE2 receptor cartoon diagram is shown in yellow and binding regions are labeled in red. Interacting amino acids are shown as stick model. **(C)** Conformational epitope as predicted by SEPIa-3 is shown in yellow color. **(D)** The ACE2 receptor is shown in a surface diagram, which has a bowl-shaped surface to accommodate loop regions of the RBD. Residues shown in red are predictive antigenic epitope and this region is shown in cartoon diagram by yellow color.

The structure based predicted antigenic epitope mostly overlaps with the predicted ACE2 receptor binding region of the RBD of SARS-CoV-2 (Figure 5). The predicted conformational epitopes are consistent with three different predicting methods. In this analysis we used both closed and open conformation of the modeled spike protein structure to predict conformational epitopes. The predictive scores for both models are very similar (Table 5). We also used 6vsb and 6vxx coordinates in structure-based antigenic epitope prediction. However, the order of prediction is changed, particularly in the RBD. The difference in the prediction is mostly due to the absence of several connecting loops in these structures, which is probably the reason for change in the predicting order. Hence, antibody production against these epitopes may be more valuable in neutralizing the viral infectivity. First, in the closed form these regions are highly solvent accessible. Secondly, in the open conformation the RBD moves away from the core region, thus there is a better chance for solvent exposure. Antibody production for these regions can possibly access the RBD both in the open as well as in the closed conformation, because these regions are solvent exposed. Furthermore, they are also involved in binding with the ACE2 receptor, which will help in decreasing the viral infectivity.

**Table 5:** Conformational predictive scores between the open and closed conformation of the RBD of spike protein of SARS-CoV-2.

**No**.	**Chain**	**Start**	**End**	**Peptide**	**Number of residues**	**Score closed**	**Score open**
1	A	469	490	**STEIYQAGSTPCNGVEGFNCYF**	22	0.838	0.805
2	A	516	526	ELLHAPATVCG	11	0.794	0.786
4	A	438	450	**SNNLDSKVGGNYN**	13	0.69	0.69
5	A	495	506	**YGFQPTNGVGYQ**	12	0.66	0.66
6	A	380	394	YGVSPTKLNDLCFTN	15	0.642	0.642
7	A	357	374	RISNCVADYSVLYNSASF	18	0.579	0.579

*Sequences represented as bold are regions that interact with ACE2 receptor*.

**Figure 6:**
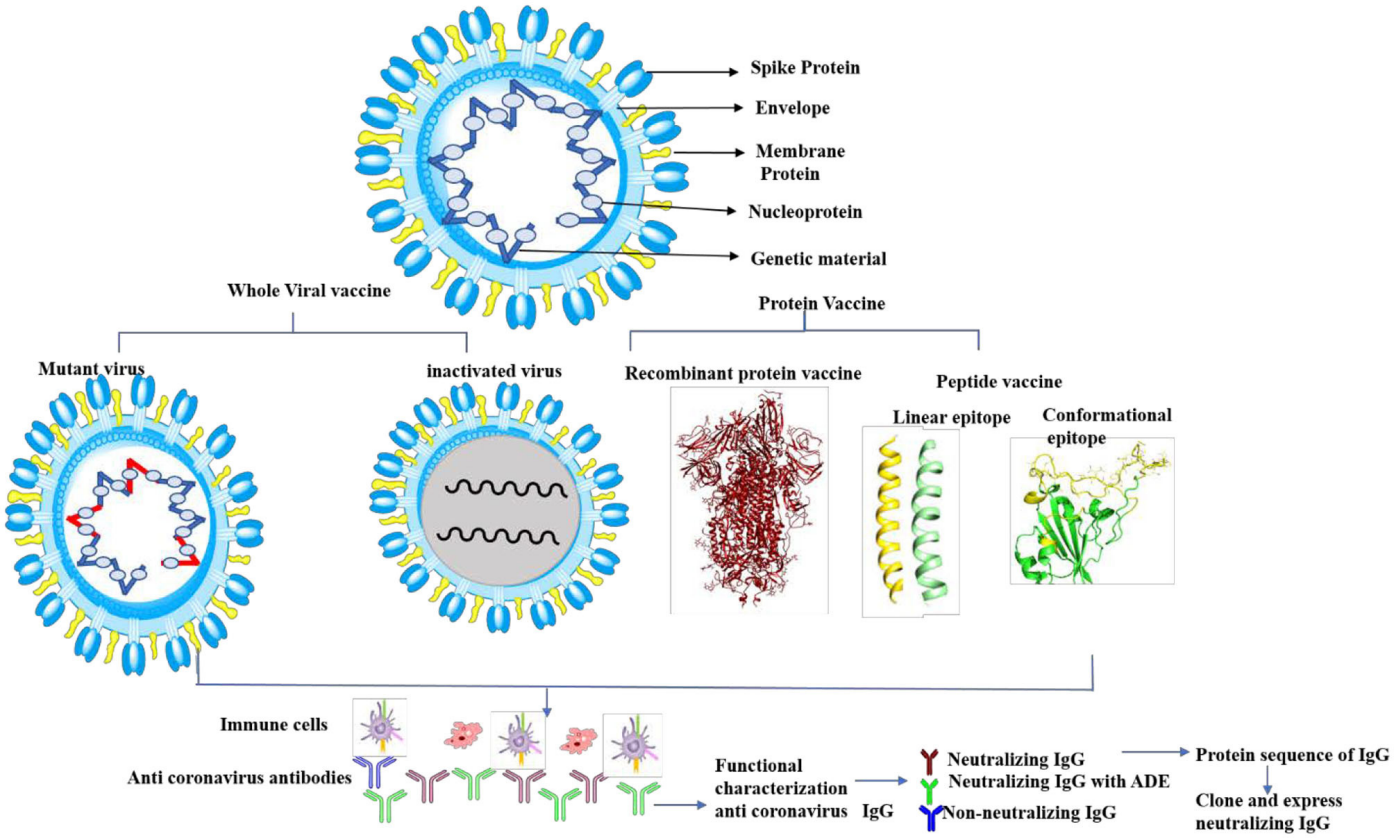
Cartoon diagram shows the proposed vaccine design by whole virus vaccine and protein-based approach. The whole virus vaccine approach, recombinant protein and peptide based antigenic epitopes can activate immune cells to produce anti-viral antibodies against SARS-CoV-2. The next step is functional characterization of these antibodies for viral neutralization properties and ADE activities. Neutralizing antibodies without ADE activity can be sequenced and cloned and expressed antibodies (with Fab and Fc region) or variable region of heavy (H) and light (L) chain to therapeutic purpose.

In conclusion, predicting antigenic epitopes based on structural information will help in producing neutralizing antibodies against the SARS-CoV-2 spike proteins, which will also help in designing vaccine strategy (Figure 6). Sterlin et al. (2021) provide evidence that SARS-CoV-2 mount specific IgA antibody at early stage infection, however, no data is available to demonstrate the longitudinal IgA response against SARS-CoV-2 in patient recovered from infection. There is a controversy regarding the effectiveness of IgA and IgG in this infection. Recent studies have shown that IgA has more potency than IgG (Ejemel et al., 2020), however, other study does not support the potency of IgA neutralization as compared to IgG (Wang et al., 2021). IgA antibodies broadly cross react with various human coronavirus (Saad-Roy et al., 2020). Secondly, the mounting and maturation of IgA occurs at early stage of infection, but IgG mounting and maturation occurs at later stage of infection (Sterlin et al., 2021). Studies also show that SARS-Cov2 specific IgA level decreases quickly in serum in Covid-19 patients (Sterlin et al., 2021). Thus, one must be very cautious while designing neutralizing monoclonal antibodies against SARS-CoV-2. A recent study shows that SARS-CoV-2 causes catastrophic microvascular injury syndrome due to the activation of the host's immune complement system (Magro et al., 2020). The immunopathological effects of antibody-dependent enhancement (ADE) have been observed in various viral infections. The same has also been reported in some COVID-19 patients (Zhang et al., 2020; Zhao et al., 2020). This group of patients had severe disease, which was characterized with a higher titer of total antibodies and an enhanced IgG response. This clearly shows an ADE-mediated viral entry and the induction of severe inflammatory responses. Thus, based on our predicted epitopes, there are opportunities to design conformational based epitopes that possibly inhibit the interaction of SARS-CoV-2 to the ACE2 receptor. Furthermore, the antigenic epitopes that lack ADE viral pathogenicity are important for viral clearance. The variable region of heavy (H) and light (L) can be also cloned and expressed without Fc region for virus neutralization, which will prevent ADE viral pathogenesis (Figure 6). Experimental evidence is needed to validate this proposed hypothesis. These specific epitopes can be also used to assess antigen-specific cell stimulation or as capture antigen to detect specific antibody responses. Thus, prediction of such epitopes with bioinformatics approaches would help to distinguish infections with common cold virus vs. SARS-CoV-2.

## Data Availability Statement

The original contributions presented in the study are included in the article/supplementary material, further inquiries can be directed to the corresponding author/s.

## Author Contributions

SK: data analysis, writing original draft, and editing. MA: reviewing and editing manuscript. PP: sequence analysis. KG: model building, antigen epitope prediction, writing original draft, and editing the final version. All authors contributed to the article and approved the submitted version.

## Conflict of Interest

The authors declare that the research was conducted in the absence of any commercial or financial relationships that could be construed as a potential conflict of interest.
